# Using a novel virtual-reality simulator to assess performance in lumbar puncture: a validation study

**DOI:** 10.1186/s12909-023-04806-z

**Published:** 2023-10-30

**Authors:** Sujun Xie, Søren Grimstrup, Leizl Joy Nayahangan, Zheng Wang, Xing Wan, Lars Konge

**Affiliations:** 1 Guangzhou University of Chinese Medicine, Jichang Road 12, Guangzhou, 510405 China; 2 Guangdong Academy for Medical Simulation (GAMS), No.10 Hongming Road, East District, Huangpu District, Guangzhou, 510530 China; 3 Copenhagen Academy for Medical Education and Simulation (CAMES), Center for Human Resources and Education, Ryesgade 53B, opg. 98A, Copenhagen, 2100 Denmark

**Keywords:** Lumbar puncture, Virtual reality, Assessment, Validity, Mastery learning

## Abstract

**Background:**

A lumbar puncture procedure’s success depends on a competent physician minimizing the risk of failing to get a sample and avoiding complications such as post-dural headache. A new virtual-reality simulator might be helpful in deciding when a physician is competent to perform lumbar puncture. We aimed to investigate validity evidence for a simulator-based test in lumbar puncture and establish a pass/fail standard to allow a mastery learning training program.

**Methods:**

Validity evidence was investigated using Messick’s framework by including participants who were novices, intermediates, or experienced in lumbar puncture. Each participant performed two lumbar puncture procedures on the simulator, and fifty-nine predefined simulator metrics were automatically recorded. Cronbach’s alpha was used to explore internal consistency reliability. Intergroup comparisons were made using independent sample t-tests with Tukey’s correction for multiple comparisons. The learning effect was explored using paired sample t-test analysis, and a pass/fail standard was established using the contrasting groups’ method.

**Results:**

73 novices, 18 intermediates, and 19 physicians performed the test resulting in a total of 220 procedures. 25 metrics (42.4%) had good discriminatory ability, and the reliability of these metrics was good, Cronbach’s α = 0.81. The experienced physicians were significantly better than the novices (18.3 vs. 13.3, p < 0.001), and the pass/fail standard was established at 16 points. This standard resulted in 22 (30.1%) novices passing (i.e., false positives) and 5 (26.3%) physicians failing (i.e., false negatives).

**Conclusion:**

This study provides validity evidence for a simulator-based test of lumbar puncture competence. The test can help ensure basic competence at the end of a simulation-based training program for trainees, i.e., a mastery learning training program.

**Supplementary Information:**

The online version contains supplementary material available at 10.1186/s12909-023-04806-z.

## Background

Lumbar puncture is a crucial procedure for diagnosing various diseases and for therapeutic purposes [1]. The success of a lumbar puncture could minimize the risk of failing to get a sample and avoid complications such as post-dural headache [2]. However, the lumbar puncture procedure can be challenging to learn, and it remains uncertain how optimal training should be arranged to ensure that trainees meet the requirements of clinical practice [3]. According to Kern’s six-step approach to curriculum development, we must be able to answer essential questions such as “How to practice?” (i.e., which educational strategy to use) and “How much to practice?” (i.e., setting goals and objectives for the training) [4].

Traditionally, medical procedures have been taught using the apprenticeship model, where novices practice directly on patients supervised by a more experienced colleague. However, ethical considerations and increased concerns for patient safety have made simulation-based training on physical phantoms and virtual reality (VR) simulators more common [5]. These modalities allow trainees to practice repeatedly in a standardized and safe environment until basic competency is acquired and they are ready for supervised practice on patients. Recent studies found good trainee satisfaction with an educational 3D video delivered in virtual reality and positive effects of hands-on training on a virtual reality lumbar puncture simulator [6, 7].

Nevertheless, how much practice is necessary? Standard courses use a fixed amount of time or a fixed number of performances. However, this approach fails to ensure competence as all trainees learn at different paces, and individual performance cannot be predicted [8]. Hence, it is strongly recommended to use Mastery Learning (ML), where each trainee continues to practice until they passes an end-of-training test. Every ML program’s success depends on the test, making it very important that it measures what it is supposed to measure, i.e., that it has solid evidence of validity [9]. Validity evidence should be gathered scientifically using a contemporary framework of validity, e.g., Messick’s framework containing five sources of evidence: Content, response process, internal structure, relationship to other variables, and consequences [10].

An assessment tool with solid evidence for validity according to Messick’s framework has already been published for lumbar puncture, the LumPAT [11]. This tool has been used to assess the performance on a physical phantom and to assess clinical procedures either by direct observation or based on video recordings of the procedure. However, experienced faculty is necessary for rating purposes, and all human assessments are prone to bias [12]. Assessments based on objective metrics provided by virtual-reality simulators have been used for other procedures to provide automatic, unbiased test results [13]. However, to our knowledge, this has not been done for lumbar puncture.

This study aimed to develop an objective and standardized test based on a newly developed lumbar puncture simulator to gather validity evidence for the test and establish a credible pass/fail standard that can ensure basic competency in lumbar puncture before continuing to clinical practice.

## Method

The development of the test and the exploration of validity was done at the Clinical Skills Center (2021–2022) at the Guangzhou University of Chinese Medicine, Guangzhou, China.

### Development of the simulator test

**Fig. 1:**
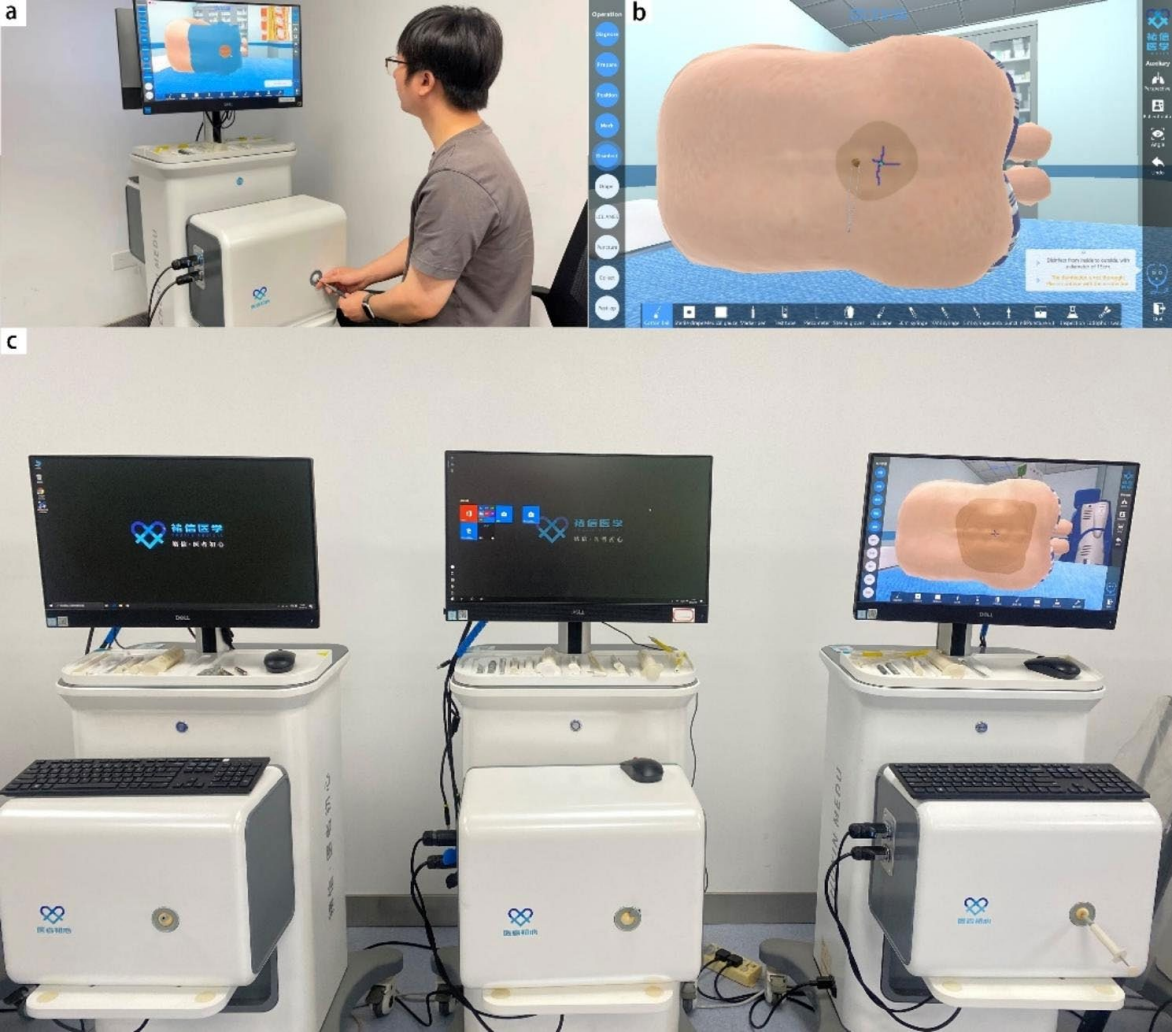
Trainee interacting with the simulator (**a**) Screen-shots from the simulator (**b**) VR simulator setup (**c**)

The Virtual Reality Lumbar Puncture simulator (Virtual Puncture Surgery Platform, CXV-CS-PVO80, Shanghai, China) consists of master controllers, pedals, and a personal display (Fig. 1). The lumbar puncture simulator delivers 59 metrics divided into 10 sub-procedures equipped with haptic and automated evaluation feedback. These are automatically recorded, ensuring unbiased outcome measures. All lumbar puncture procedures in the simulator were tested by an expert (who had performed more than 500 lumbar punctures), who chose a typical case of a 52-year-old male who presented with a headache for six days and was admitted to the neurology department.

### Participants in the validation study

Participants were novices, intermediates, and experienced physicians. They were recruited through campus network notification and WeChat groups. Novices were medical students in years 3–4 from the Guangzhou University of Chinese Medicine without previous lumbar puncture training. Intermediates were residents from various affiliated hospitals of Guangzhou University of Chinese Medicine who had taken a lumbar puncture course using a phantom and had performed 1–3 lumbar punctures on patients. Experienced physicians were doctors who had performed more than 50 lumbar punctures including the neuraxial procedures such as subarachnoid blocks, epidurals, and lumbar drain placements. They came from different neurosurgery departments, departments of internal medicine (including neurology, emergency care unit), and anesthesiology at the First, Second, and Third Affiliated Hospital of Guangzhou University of Chinese Medicine and had taken part in the lumbar puncture simulation-based curriculum.

### Administration of the test

A 5-minute video illustrating a virtual reality simulation of a lumbar puncture was shown to each participant before the test. Then each participant performed a lumbar puncture procedure on the simulator. A simulator operator was available for assistance for any technical issues but not procedural advice. After the first test, the participants received feedback from automatic metrics provided by the simulator, then the participants repeated the same lumbar puncture procedure a second time.

### Statistical analysis

Internal consistency reliability was calculated using Cronbach’s Alpha to explore the consistency of the scores across the different items in the test. An item analysis was performed to calculate Item Difficulty and Item Discrimination Index, according to the recommendations of Thomas Haladyna [14]. The final test consisted of the items with an appropriate level of difficulty and good discriminatory ability. The relationship to other variables was calculated by comparing the scores of the three groups using independent sample t-tests with Tukey’s correction for multiple comparisons. The learning effect was calculated by paired sample T-test. Finally, the contrasting groups’ methods were used on novices and experienced physicians to establish a pass/fail standard [15]. The consequences of this standard were reported by the numbers of false positives (novices that passed the test) and false negatives (physicians that failed the test) and by using Fisher’s exact test to compare these results. All statistical analyses were done using IBM SPSS Statistics version 28. P-values less than 0.05 were considered statistically significant.

## Results

A total of 110 participants were included in the study, and all performed two simulated lumbar punctures procedure. Table 1 shows the group allocation, experience level, and participants’ demographics.

**Table 1:** The demographics and experience of the three groups

Group	Gender men/women	Age Mean (min-max)	Lumbar punctures performed	First test Total Test Score Mean (SD)	Second test Total Test Score Mean (SD)
Novices (n = 73)	22/51	21.0 (20–22)	0	11.2 (4.0)	13.3 (4.3)
Intermediates (n = 18)	6/12	24.4 (23–28)	1–3	15.9 (3.3)	15.5 (3.7)
Physicians (n = 19)	7/12	40.4 (27–50)	> 50	15.8 (6.4)	18.3 (4.7)

The item statistics analysis showed that 27 out of 59 items had a difficulty index between 0.25 and 0.91, i.e., an appropriate level of difficulty (not extremely easy or hard). All but two of these items also had a good discriminatory ability above 0.10, resulting in 25 out of 27 items being included in the final test. Six of these metrics (24%) were diagnostic, five items (20%) concerned the preparation of the procedures, two (8%) were regarding the identification of landmarks, 10 (40%) tested skills in disinfection, and the last two (8%) tested local anesthesia skills. (Table 2).

**Table 2:** The item difficulty index (Item diff) and item discrimination index (Item disc) of the 25 items in the final test

Item	Sub-procedure	Item details	Item diff	Item disc
2	Diagnosis	Checks for the medical history	0.25	0.30
3	Diagnosis	Checks for the patient’s physical examination	0.36	0.41
4	Diagnosis	Checks for the auxiliary examination	0.31	0.42
5	Diagnosis	Judges the performance correctly	0.29	0.40
6	Diagnosis	Improper judgment of the performance (or missing out)	0.38	0.31
7	Diagnosis	Considers contraindications	0.66	0.17
10	Preparation	Prepares environment	0.27	0.52
12	Preparation	Prepares patient	0.54	0.45
14	Preparation	Prepares himself/herself	0.85	0.13
17	Preparation	Prepares material	0.38	0.21
20	Preparation	Checks the puncture kit and the expiry date	0.65	0.19
22	Marking	Marks site appropriately and precisely	0.82	0.35
23	Marking	Does not mark the puncture site	0.86	0.28
25	Disinfect	Misses an area or slightly offset for the first disinfection	0.68	0.18
26	Disinfect	The second disinfection is correct	0.27	0.32
27	Disinfect	Misses an area or slightly offset for the second disinfection	0.4	0.28
28	Disinfect	The third disinfection is correct	0.38	0.47
29	Disinfect	Misses an area or slightly offset for the third disinfection	0.45	0.48
30	Disinfect	Appropriate frequency of disinfecting	0.84	0.40
31	Disinfect	Opens the puncture kit in the right way	0.35	0.44
32	Disinfect	Ensures the disinfection quality of the puncture kit	0.28	0.45
33	Disinfect	Wears the gloves correctly	0.49	0.33
34	Draping	Lays surgical drape correctly	0.89	0.22
35	Local anesthesia	Checks the lidocaine	0.54	0.36
36	Local anesthesia	Selects the 5 ml syringe	0.58	0.37

### Response process

Validity evidence regarding this source was ensured by standardizing the testing process: All tests were facilitated by the same three experienced simulator operators who did not offer any procedural advice during the tests. Potential bias was eliminated by using the automatic simulator judgment.

### Internal structure

**Fig. 2:**
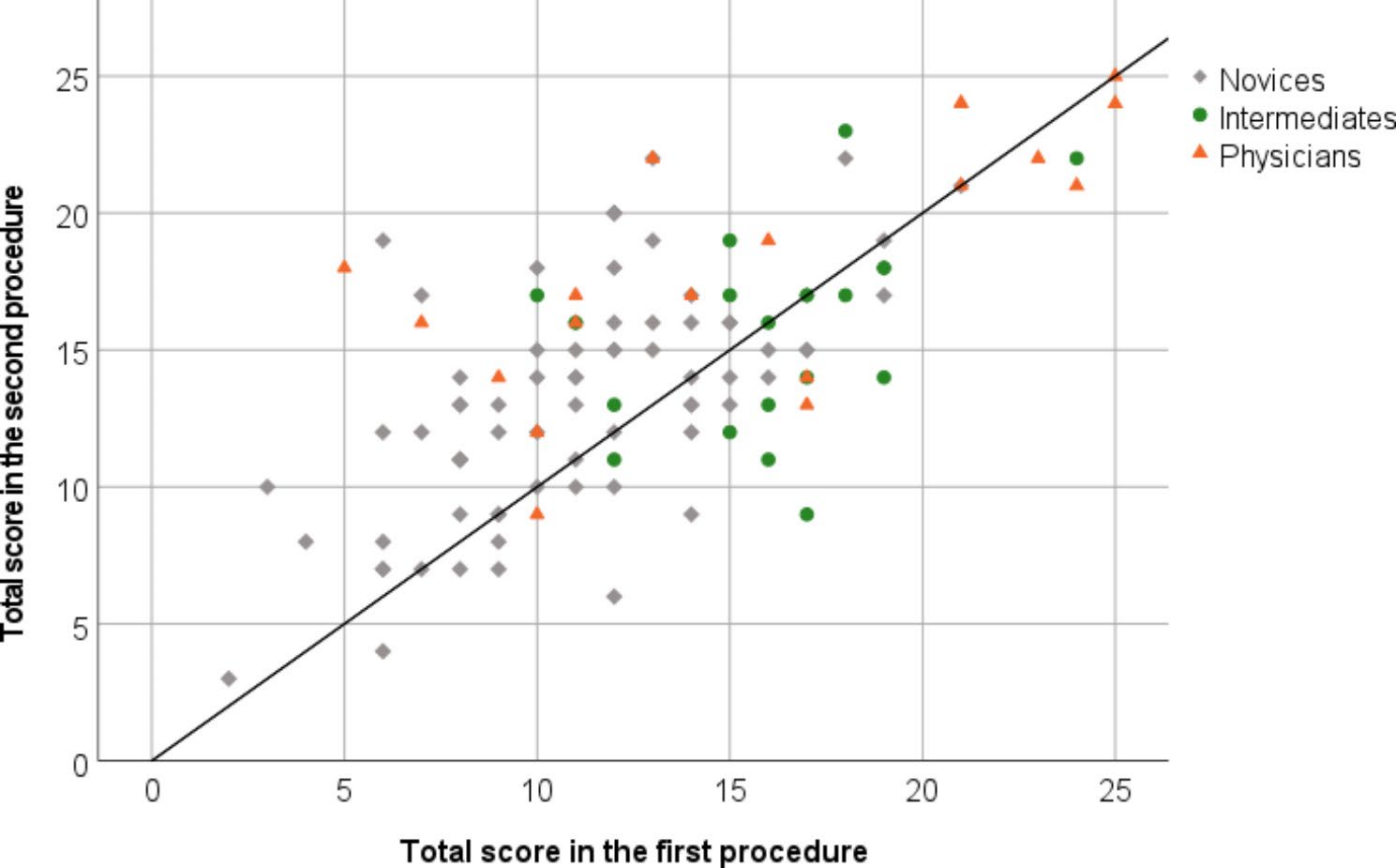
Scatter Plot of Total Score by Groups

The internal consistency for the 25 included simulator metrics was 0.81, CI 95% [0.76–0.86]. A Pearson’s correlation of r = 0.66 [0.54,0.76], p < 0.001 demonstrates a highly significant and relatively strong correlation between the 1st and 2nd test (Fig. 2).

### Relationship to other variables

The relationship to other variables was explored by comparing the scores of the three groups using independent sample t-tests with Tukey’s correction for multiple comparisons for test 1 and test 2 separately (Table 3). The experienced physicians performed significantly better than the novices in both procedures.

**Table 3:** Multiple Comparisons of total score between groups

	Group (a)	Group (b)	Mean difference (a-b)	P value	95% CI	
					Lower Bound	Upper Bound
First procedure	Novices	Intermediates	-4.71	< 0.001	-7.46	-1.96
	Intermediates	Experienced	0.15	0.99	-3.28	3.59
	Experienced	Novices	4.56	< 0.001	1.87	7.25
s procedure	Novices	Intermediates	-2.17	0.11	-4.84	0.49
	Intermediates	Experienced	-2.82	0.12	-6.15	0.51
	Experienced	Novices	4.99	< 0.001	2.38	7.59
Mean test score	Novices	Intermediates	-3.44	0.003	-5.87	-1.01
	Intermediates	Experienced	-1.33	0.55	-4.37	1.71
	Experienced	Novices	4.77	< 0.001	2.39	7.15

Independent sample t-tests with Tukey’s correction for multiple comparisons

### Consequences

**Fig. 3:**
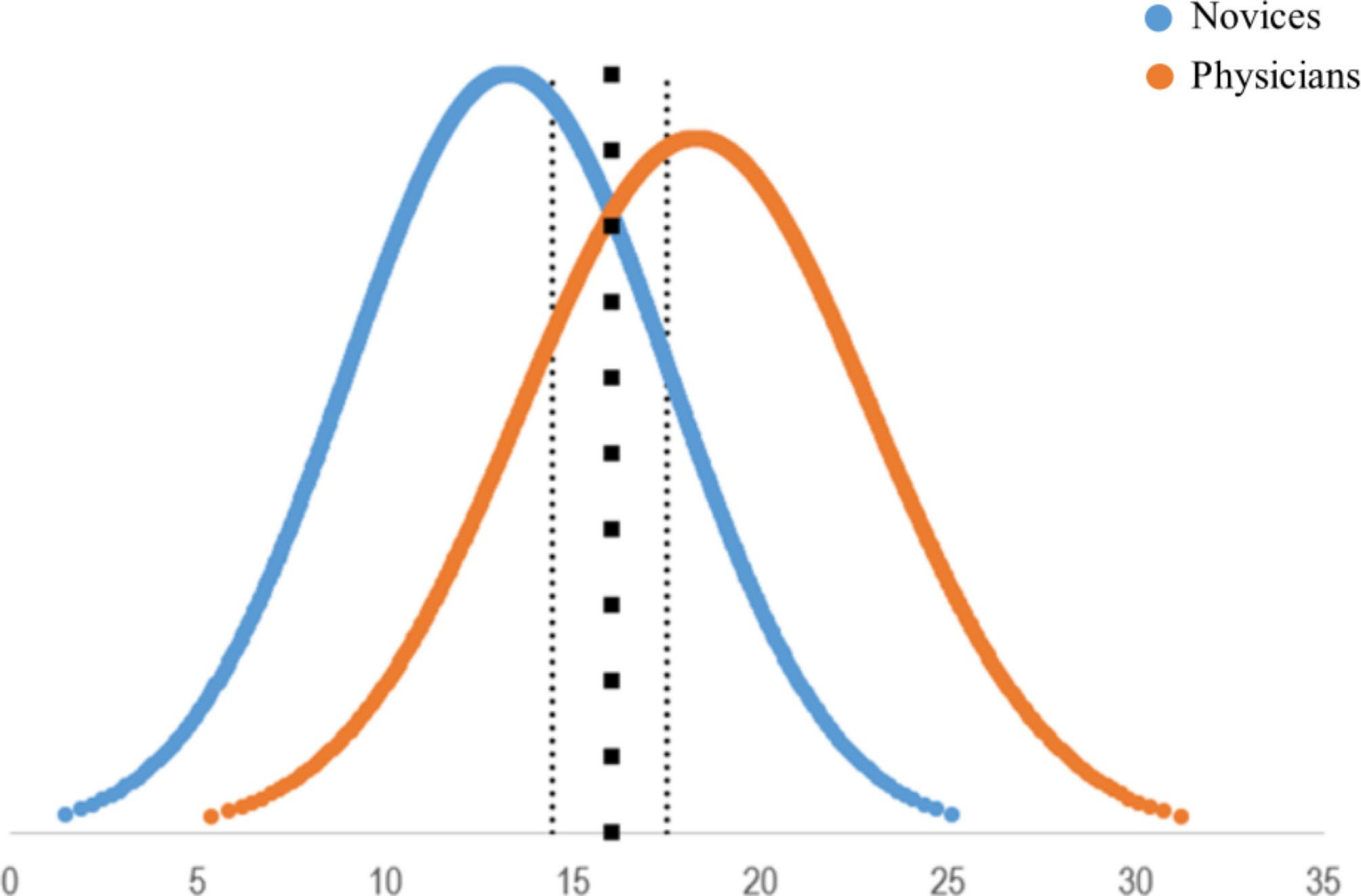
Establishment of the pass/fail standard using the contrasting groups’ method

A pass/fail standard was established at 16 points, CI 95% 14.4–17.5 points, Fig. 3. This standard resulted in 22 (30.1%) novices passing (i.e., false positives) and 5 (26.3%) physicians failing (i.e., false negatives).

### Learning effect

**Fig. 4:**
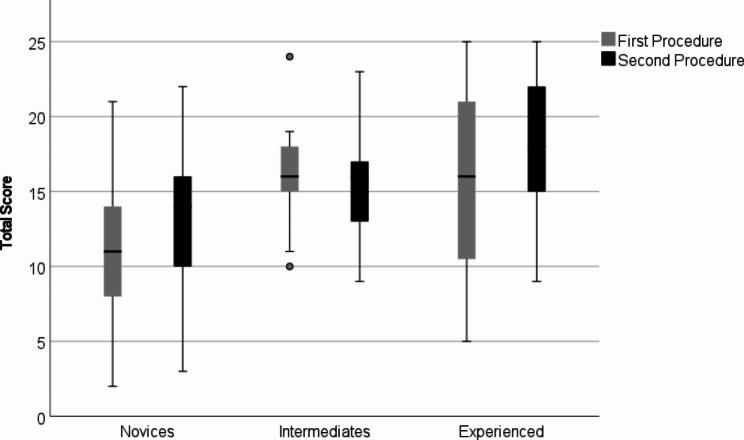
Total sum of 25 items in the first and second procedure between different groups

Comparing the results from the first and the second procedure with paired sample t-tests showed that the novices and the experienced physicians improved significantly in the total score. However, the intermediates did not improve significantly (Fig. 4; Table 4).

**Table 4:** Difference between 1st and 2nd procedure

Group	N	mean		95% CI	
			p-value	Lower Bound	Upper Bound
Novices	73	2.1	< 0.001	1.26	2.93
Intermediates	18	-0.44	0.64	-2.41	1.52
Physicians	19	2.53	0.03	0.33	4.72
Total	110	1.75	< 0.001	1.02	2.49

## Discussion

In this study, we developed a new virtual reality simulation-based test of competence in the lumbar puncture procedure. One-hundred and ten medical students and physicians took the test in a standardized setting, and solid evidence of validity was established for all five sources in the contemporary validity framework of Messick [16]. This research is the first validity study using Messick’s five sources to explore a test based on a VR simulator for lumbar puncture with haptic feedback.

### Internal structure

The internal consistency of the 25 items was good, with Cronbach’s alpha = 0.81. High-stake tests, e.g., end-of-course or end-of-year summative exams in medical school, need a reliability of more than 0.8, making our test suited for mastery learning training programs. The Lumbar Puncture Assessment Tool (LumPAT) had an internal consistency of 0.92 but relied on expert ratings, which could introduce issues concerning subjectivity and bias. A study on infant lumbar puncture used residents as raters and found an acceptable internal consistency of 0.77 [17]. Ma et al. explored an error-focused checklist in lumbar puncture and found a low internal consistency of 0.35. Despite this relatively low reliability, they still recommend using the error-focused checklist to identify procedural incompetence [18]. It could be worth exploring whether a combination of our objective test of competence could be combined with an error-focused checklist to better identify the superior and safe performance of competent trainees.

### Relationship to other variables

Experienced participants performed significantly better than novices in both procedures. However, physicians performed about the same as the intermediates, which may indicate that the simulator cannot discriminate the small nuances in the lumbar puncture procedure, a problem also reported with a VR simulator for robot-assisted radical prostatectomy [19]. However, our simulation-based test possessed discriminatory ability as opposed to a test using a virtual reality simulator of uretero-nephroscopy, which could not even discriminate between novices and experts [20].

### Consequences

We used a recommended standard-setting method to establish a pass/fail limit of 16 points. Unfortunately, there was a considerable variation in performances, and 26.3% of the experienced physicians failed the test. As they were unfamiliar with the virtual reality simulator, a longer warm-up (i.e., a familiarization phase) could solve these issues. Gustafsson et al. used a VR simulator to explore the learning curves of orthopedic surgeons. They found that experienced surgeons needed to perform seven simulated hip fracture procedures before they performed in a way that resembled their actual competence [21]. Warm-up on a simulator is a good idea in research on SB training and could positively impact the real clinical world. Chen et al. found that performing a brief warm-up exercise before a laparoscopic procedure significantly improved the intraoperative performance of residents [22]. Future studies using the VR lumbar puncture simulator should investigate the learning curves of both trainees and the familiarization curves of experienced physicians.

### Virtual reality simulation or simple phantoms?

VR is an emerging technology that creates a virtual environment for users to get an aesthetic feel for the desired surroundings [23]. In this study, novices got a higher score on the second test (2.1 points improvement, *p*<0.001), indicating that the VR simulator’s automatic feedback is valuable when training. VR simulators offer several kinds of automatic feedback, which encourage the trainee to practice again to achieve or meet the required level [24]. However, the simulators often come at a high cost. They should only be integrated into a well-thought-out training program, e.g., mastery learning programs using evidence-based pass/fail standards [25]. Physical phantoms are less expensive but require direct observation by expert instructors, which is both time-consuming and expensive [26]. Our study makes it possible to implement a mastery learning program where novices practice on the simulator while receiving automatic evaluations and structured feedback after each performance. Simulation-based training can accelerate the trainees’ learning curves [27]. However, future studies must explore the transfer of skills to procedures on actual patients after trainees have trained to our predefined mastery level.

### Limitations

Our study has several limitations. First was the heterogeneous background of experienced physicians from several different specialties. They were recruited because they were key teaching staff of lumbar puncture, but several did not perform the actual procedure regularly. The attainment and maintenance of a 90% success rate may require 45–60 attempts at spinal and epidural anesthesia [28]. The neurosurgical doctors have a high lumbar puncture activity, but unfortunately, it was not easy to recruit many of these.

Secondly, the final test is unbalanced. Many items probed disinfection technical issues (40% of items). In contrast, few items relating to the actual puncture were included in the final test which may explain why some of the included novices managed to pass the test with very little lumbar puncture experience. The current version of the simulator has limitations concerning the tactile sensation during the needle insertion. Vamadevan et al. report that haptic virtual reality simulators reduce the time to reach proficiency compared to the non-haptic simulator. However, the acquired skills are not transferable to the conventional non-haptic setting [29]. In the future, the haptic feedback of the VR simulator should be improved and allow more specific items regarding the actual procedure to be included in the test. Alternatively, the learning process on the simulator should be supplemented by needle punctures on physical models. This could make the test better at measuring the actual puncturing skills which would probably reduce the number of false positives, i.e. novices that manage to pass the test without adequate skills.

## Conclusion

Our study provides validity evidence for a virtual reality simulator-based test of lumbar puncture competence. We established a pass/fail level, which can be used to make a mastery learning training program without the need for expert faculty.

### Electronic supplementary material

Below is the link to the electronic supplementary material.

Supplementary Material 1

Supplementary Material 2

## Data Availability

The datasets used and/or analyzed during the current study are available from the corresponding author upon reasonable request.
