# Supplementary material for: Using a novel virtual-reality simulator to assess performance in lumbar puncture: a validation study
Source: BMC Med Educ. 2023 Oct 30;23:814. doi: 10.1186/s12909-023-04806-z (PMC10614418; doi:10.1186/s12909-023-04806-z)
Supplement: Supplementary file 2 — Supplementary Material 2 [file 12909_2023_4806_MOESM2_ESM.docx]

Demonstration Video

Title: Lumbar puncture procedure training on the VR device

Legend: By touching the screen, the trainees made a diagnosis of indications and contraindications for lumbar puncture based on the case information, then started the lumbar puncture procedure, and the simulator mimics the sensation of puncture when performing local anesthesia and puncture.
